# Bilateral Calcified Subdural Empyemas: A Case Report and Literature Review

**DOI:** 10.7759/cureus.79704

**Published:** 2025-02-26

**Authors:** Veronica A Colmenares, Sarah Danehower, Brian J Williams

**Affiliations:** 1 Neurosurgery, University of Louisville School of Medicine, Louisville, USA; 2 Neurosurgery, University of Louisville Hospital, Louisville, USA

**Keywords:** craniotomy, dural calcification, infection, rare condition, subdural empyema

## Abstract

Calcified subdural empyemas (CSEs) are rare neurosurgical pathologies that can be mistaken for calcified chronic subdural hematomas. Patients often present with symptoms of worsening headache, seizures, or hemiparesis without general malaise or systemic illness. Here we report the tenth documented case of CSE in a 51-year-old man from South Africa with no surgical history who developed bilateral, culture-positive collections. Additionally, we provide a review of the current literature on this condition. The majority of CSEs occur in males from developing countries who previously underwent neurosurgical procedures decades earlier. Further investigations into the pathophysiology of this unique disease are warranted.

## Introduction

Calcified subdural empyemas (CSEs) represent a rare and potentially life-threatening neurosurgical condition [1]. To date, there have only been eight case reports describing the clinical course of these lesions [2-8]. Furthermore, each case features varied patient demographics and presentations, symptomatology, and surgical treatment. Given the rarity of presentations and the lack of consensus on treatment, patient management is left entirely to individual providers. Herein, we present a case of bilateral CSEs and review the current literature. 

## Case presentation

**Figure 1:**
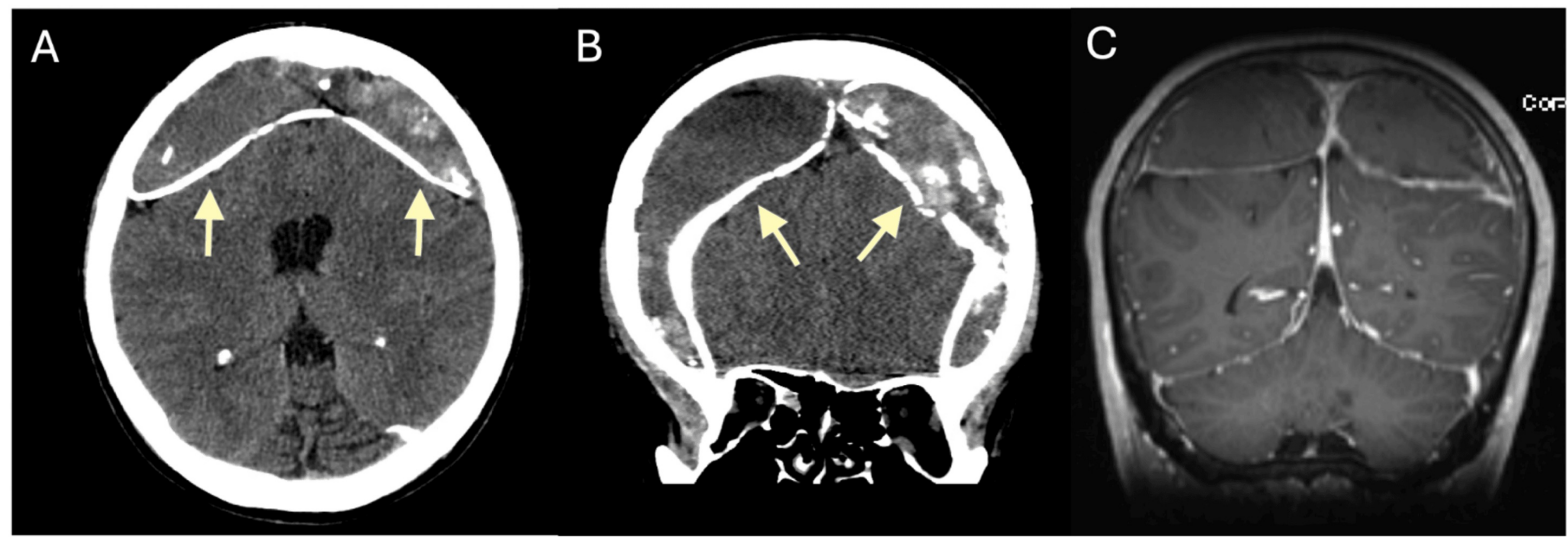
(A) Axial CT with bilateral calcifications (arrows). (B) Coronal CT revealing sulcal effacement and mass effect (arrows). (C) Coronal MRI with contrast with no enhancement of the collections.

Our patient, a 51-year-old man from South Africa, presented with headaches refractory to over-the-counter medications. The patient stated he fell while farming about seven months prior to visiting the hospital but denied further trauma or symptoms. He had no significant medical history of meningitis, fever, or prior surgeries. The patient was neither on antiplatelet medications nor immunocompromised. Physical examination revealed he was neurologically intact and vital signs were stable. He was afebrile on presentation. Computed tomography (CT) was obtained which revealed bilateral, calcified extra-axial collections (Figure 1A-1B). MRI with contrast was also obtained to better characterize the collections (Figure 1C). 

The collections were diagnosed as acute-on-chronic subdural hematomas as there was no pathologic enhancement or diffusion restriction noted. Given the patient’s symptoms and the mass effect seen, surgical evacuation was planned.

**Figure 2:**
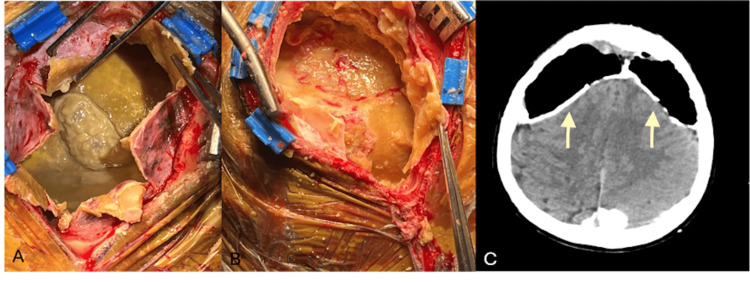
(A) Intraoperative images of left craniotomy and right craniotomy with purulent material. (B) Intraoperative images of left craniotomy and Right Craniotomy after evacuation of purulent material. (C) Post-operative axial CT revealing empyema evacuation and retained calcified membranes with resulting pneumocephalus and no resolution of sulcal effacement (arrows).

Bilateral mini craniotomies were performed. The extensively calcified dura was opened in a cruciate fashion with immediate return of purulent appearing material under pressure. The opposite side was incised with comparable results. Evacuation of the purulent material was limited by the exposure, draining veins traversing the space and extent of the pathology (Figure 2A-2B). Cultures were sent during surgery and empiric antibiotics were given. Post-operatively, while awaiting culture results, the patient was started on intravenous vancomycin and cefepime. He was later transitioned to intravenous ceftriaxone. Once the infectious material was evacuated, it was noted that the brain remained compressed underneath the calcified membranes. However, these membranes were adherent to the brain and the decision was made to leave them in situ. The remaining subdural space was filled with irrigation and the craniotomies were closed in a standard fashion with subdural drains.

Post-operative imaging revealed evacuation of collections but no resolution of sulcal effacement given the known calcifications (Figure 2C). Clinically, the patient remained stable with continued headaches. Cultures were positive for *Staphylococcus epidermidis *(*S. epidermidis*), and the patient was treated with an eight-week course of nafcillin.

Six months after the patient’s first surgery he presented with left incisional drainage. Given the persistent drainage, the patient was taken for wound washout. Intra-operative cultures revealed* S. epidermidis* and the patient was started on a second regimen of nafcillin per infectious disease recommendations. The patient tolerated the procedure well and was discharged home in stable condition. He reported continued headaches but denied seizures, nausea, and vertigo at the last follow-up.

## Discussion

A literature search for CSEs yielded eight results with nine cases, making our case the tenth (Table 1). One report described two cases but was excluded, given the inaccessibility of the manuscript [9]. Of the eight cases, six (75%) of the patients were male and six (75%) had cultures that never produced an infectious etiology. Five of the patients had undergone previous neurosurgical procedures that were the suspected etiology of their CSE, with ventriculoperitoneal shunts being the most common etiology (3 out of 5; 60%). Seven of the eight cases occurred in developing countries, with three occurring in India. Two patients died due to complications from surgical evacuation.

**Table 1:** Summary of Documented Case Reports

Author, Year	Patient Age	Sex	Suspected cause	Culture results	Major symptom	Patient outcome	Country of origin
Sarkar et al., 2012 [2].	11	M	Meningitis	Aseptic	Seizures	Reduced seizure burden	Bangladesh
Bhardwaj et al., 2020 [3].	25	M	Meningitis and ventriculitis from ventriculoperitoneal shunt placement at age 4	Aseptic	Open wound with drainage, headache	Resolution of wound	India
Nour and Shumbash, 2020 [4].	39	M	Unknown	Aseptic	Hemiparesis	Death due to tension pneumocephalus	Ethiopia
Kaspera et al. 2005 [5].	47	F	Subdural empyema that occurred 46 years prior from otitis media	Aseptic	Seizures	Decreased seizure burden	Poland
Beker-Acay et al., 2016 [6].	2	M	Ventriculoperitoneal shunt placed 1 year prior	Achromobacter denitrificans	Seizures	Death	Turkey
Kasliwal et al. 2009 [7].	17	F	Ventriculoperitoneal shunt placed 9 years prior	Aseptic	Hemiparesis	Improved strength	India
Verma et al., 2018 [8].	44	M	Cranioplasty after craniectomy for subdural hematoma evacuation after trauma	Aseptic	Wound drainage	Resolution of wound	India
Present Case	50	M	Unknown	Staphylococcus epidermidis	Headache	Revision surgery for wound drainage	South Africa
Summary	Median age: 32	Sex predominance: Male	Most common etiology: ventriculoperitoneal shunt	Most common pathogen: aseptic	Most common presenting symptom: seizures	Most common treatment outcome: symptom improvement	Most common country of origin: India

Presently, there is a paucity of literature on CSEs. Of the handful of case reports in the literature, there is a lack of consensus on patient demographic risk factors, presentation, and management of this complex pathology. In contrast to typical subdural empyemas which present with acute neurological decline including seizures, fevers, and severe systemic illness, patients with CSEs often present with nebulous complaints including headaches, seizures, and weakness for months to years [10, 11]. Only upon further workup with CT and MRI is a calcified, non-enhancing collection with local mass effect revealed. Given the lack of enhancement, these collections are often misdiagnosed as chronic subdural hematomas, which are only rectified in the operating room with direct visualization of extensive purulence. 

Our patient’s initial intraoperative culture was positive for rare growth of *S. epidermidis* which was suggested to be contamination or colonization. *S. epidermidis* is part of the normal skin and mucosa microbiota. In most cases, it is considered a commensal organism, but it can be involved in nosocomial infections [12]. Its pathogenicity stems from the ability to form biofilms resistant to antibiotics. As such, most people with* S. epidermidis* infections have implanted devices that require surgical removal for source control. Our patient did not have an implanted device or previous surgery that would explain the intracranial presence of *S. epidermidis*. Therefore, in an abundance of caution, the patient was maintained on antibiotics to treat the possibility of central nervous system infection. 

Our literature review yielded only one other case report with an identified infectious etiology, *Achromobacter denitrificans*. This patient’s presentation and demographics were unique compared to the aseptic population [6]. The patient was significantly younger than others, and his clinical decline occurred more rapidly over one year. Similarly, our patient experienced a progressive clinical decline over a year following minor trauma. Therefore, culture-positive CSEs may represent a unique subset of this population with a more aggressive course. 

Literature review revealed the majority of CSE etiologies to be aseptic infections following previous neurosurgical interventions [4, 6, 13]. However, our patient had never undergone neurosurgical intervention or experienced neurological infection. He reported minor cases of otitis media as a child but denied requiring hospitalization or medical treatment for these infections. However, given the patient was from South Africa, a developing country, his access to medical care was limited. In fact, all but one of the reported CSE cases occurred in developing countries in which access to healthcare is limited. As such, most patients reported prolonged symptoms prior to presentation and identification of CSE. Not only may these patients be exposed to atypical pathogens which lead to indolent infections, but their access to proper treatment may be limited, resulting in delayed identification and treatment. Therefore, careful consideration should be given to patients presenting with chronic neurologic complaints who are from developing countries and have undergone previous neurosurgical procedures. 

## Conclusions

CSEs represent a rare pathology typically diagnosed after a prolonged clinical course. Unlike subdural empyemas, these collections are typically aseptic, and patients present with milder symptomology. Nevertheless, CSEs are associated with serious morbidity and mortality, and prompt diagnosis and treatment are needed. Further efforts to elucidate the etiologies of this unique pathology are warranted. 
